# Emergency planned re-infusion therapy and hospitalisation for community-acquired pneumonia: a retrospective case-control study

**DOI:** 10.7189/jogh.15.04044

**Published:** 2025-01-31

**Authors:** Sheng-Xian Chen, Zhi-Kai Yang, Lin Lin, Hou-Zhen Liao, Xiao-Ting Xiang, Di Liu, Jian-Shan Huang

**Affiliations:** 1 Department of Emergency Clinic, Xiamen Chang Gung Hospital, Xiamen, China; 2 Department of General Medicine, Xiamen Chang Gung Hospital, Xiamen, China

## Abstract

**Background:**

Community-acquired pneumonia (CAP) is a frequent reason for emergency department visits and leads to increased direct medical costs, particularly due to hospitalisation. This study aims to examine the differences between emergency planned re-infusion therapy and hospitalisation in patients with CAP.

**Methods:**

This retrospective case-control study involved 1889 CAP patients treated at the Emergency Internal Medicine Department of Class A tertiary Hospital in China from 1 January 2020 to 31 December 2022. Patients were divided into groups receiving either emergency planned re-infusion therapy or hospitalisation. Independent sample *t* tests and χ^2^ tests were used to compare the clinical outcomes and economic impacts between the two groups across different pneumonia severity index (PSI) classifications.

**Results:**

The study enrolled 1889 CAP patients. For PSI I–II patients, the improvement rates were 99.51% in the emergency planned re-infusion therapy group and 99.69% in the hospitalisation group, showing no statistically significant difference (*P* > 0.05). Similarly, no significant difference was observed for PSI III patients (84.16 vs. 89.82%). However, significant differences emerged for PSI IV patients, with improvement rates of 50% in the emergency planned re-infusion therapy group and 90.59% in the hospitalisation group (*P* < 0.001). Statistically significant differences were also noted in treatment duration (5.13 ± 1.65 days vs. 7.60 ± 3.93 days, *P* < 0.001) and total treatment costs (1921.57 Chinese Yuan (CNY) ± 923.16 vs. 9083.80 CNY ± 3583.55, *P* < 0.001) between the two groups.

**Conclusions:**

Emergency planned re-infusion therapy for CAP is an effective and cost-efficient alternative that can reduce both treatment duration and costs, particularly for patients with PSI I–III. It is recommended that emergency physicians give priority to emergency re-infusion therapy for patients with PSI I–III. In addition, it is recommended that hospitals strengthen the classification and treatment training programmes for emergency department physicians to recognise the patients with PSI I–IV.

**Registration:**

The hospital^’^s ethics committee (XMCGIRB2024034-01)

Community-acquired pneumonia (CAP) is a prevalent and serious health issue globally. Despite advancements in effective antimicrobial therapies, CAP still significantly impacts emergency departments and contributing to high morbidity and mortality rates [1,2]. According to the WHO Global Burden of Disease study, lower respiratory tract infections, including CAP, rank as the fourth leading cause of death worldwide, causing approximately 429.2 million episodes of illness annually [3,4]. In 2021, the reported average pneumonia mortality rate in China was 11.22 per 100 000 [5]. In addition to its health implications, CAP is among the most costly conditions requiring hospitalisation, with total hospital costs in the USA estimated at 6.4 billion USD in 2017 [6,7]. Clinicians typically classify CAP patients into five risk categories based on the community-acquired pneumonia severity index (PSI), which correlates with different 30-day mortality rates ranging from 0.1% in class I to 29.2% in class V [8]. Recommended treatments vary: patients with PSI I–II generally receive outpatient oral antibiotics; those with PSI III–IV are usually hospitalised; and PSI V patients often require intensive care unit (ICU) admission. However, significant variability exists in hospitalisation rates for CAP, suggesting that clinicians' decisions on hospitalisation may not be uniform and may often overestimate the risk of death [9,10]. Furthermore, many patients prefer alternatives to hospitalisation when given the choice [11].

During the 2019–2022 COVID-19 pandemic, some patients with CAP who tested negative for the COVID-19 nucleic acid had to opt for planned re-infusion therapy due to the strain on and shortage of beds and health care resources. Additionally, some patients chose this therapy voluntarily because of financial constraints or fear of cross-infection during hospitalisation. It was also observed that there was a decrease in the number of CAP hospitalisations during the COVID-19 pandemic, particularly among patients with mild disease [12,13]. To date, few studies have analysed the clinical efficacy and economic burden of CAP patients treated with planned re-infusion therapy in the emergency department. Understanding the differences between emergency planned re-infusion therapy and hospitalisation for patients with CAP is crucial in improving and establishing a process for emergency planned re-infusion therapy.

## METHODS

### Study participants

General information patients who visited the Emergency Internal Medicine Department of Class A tertiary Hospital in Xiamen between 1 January 2020 and 31 December 2022, were selected as study participants. The study protocol was approved by the hospital^’^s ethics committee (XMCGIRB2024034-01). These patients were diagnosed with new inflammatory infiltrates by imaging such as computed tomography (CT)or chest films and tested negative for the novel coronavirus. Inclusion criteria were as follows:

1) diagnosed with CAP by lung imaging accompanied by fever and respiratory symptoms

2) tested negative for the novel coronavirus

3) aged between 18–80 years

4) consented to undergo emergency planned re-infusion therapy or hospitalisation

5) diagnosed with CAP of PSI I–IV and treated in the general ward.

Exclusion criteria included:

1) age under 18 or over 80 years

2) positive test result for the novel coronavirus

3) treatment with oral medication only

4) refusal of emergency planned re-infusion therapy, interruption of treatment, lack of clinical information, or refusal of hospitalisation

5) diagnosis of CAP of PSI V and/or admission to the ICU

6) patients from nursing homes with hospital-acquired pneumonia.

Trained professionals collected the data and trained professionals examined the data and eliminated subjects with incomplete information (such as missing data)

Patients with CAP were divided into two groups: those receiving emergency planned re-infusion therapy and those undergoing hospitalisation.

### Treatment method

1. Definition and methods of emergency planned re-infusion therapy: Definition of ‘emergency planned re-infusion therapy’: emergency patients with a diagnosis of CAP are scheduled to return to the emergency department daily for infusion therapy as directed by the emergency physician. Method: emergency physicians empirically selected appropriate intravenous antibiotics, such as cephalosporins (ceftriaxone, cefoperazone, and sulbactam), quinolones (levofloxacin, moxifloxacin), and macrolides (azithromycin) based on disease severity, supplemented by oral medications for cough suppression, sputum removal, and fever reduction. Patients were informed of the time to return for daily infusions and underwent routine blood examinations every three days until significant symptom relief or hematological improvement was achieved. Post-infusion, patients were transitioned to oral antibiotics and scheduled for a follow-up in the respiratory clinic after one week.

2. Hospitalisation method: Pulmonologists provided standardised treatment based on the severity of the disease, adhering to the Chinese community-acquired pneumonia guidelines [14]. Treatment effect assessment criteria should refer to the Chinese community-acquired pneumonia guidelines [14]. The criteria for assessing treatment efficacy were based on the Chinese community-acquired pneumonia guidelines:

1) clinical cure and normalisation of inflammatory indices were the standards

2) significant relief of general conditions (included body temperature ≤37.8°C, heart rate ≤100 beats/min, respiratory rate ≤24 breaths/min, systolic blood pressure ≥90 mm of mercurymm (Hg), oxygen saturation ≥90% or arterial oxygen pressure ≥60 mm Hg on room air)

3) significant relief of clinical symptoms such as cough, expectoration, chest tightness, chest pain, and dyspnea; return of pulmonary auscultation respiratory sounds to normal.

### Statistical analysis

All data were statistically analysed using SPSS version 22.0 (IBM Corp., Armonk, NY, USA). Continuous variables that conformed to a normal distribution were presented as mean ± standard deviation (τ̅ ± s), and comparisons between the two groups were conducted using the independent sample *t* test. Categorical variables were expressed as percentages and compared using the χ^2^ test or Fisher exact test. The analysis focused on differences between CAP patients in the emergency planned re-infusion therapy group and the hospitalisation group in terms of age, gender, vital signs, test results, underlying diseases, treatment duration, treatment costs, and prognosis. Logistic regression analysis was employed to identify factors influencing the hospitalisation of CAP patients, with a *P*-value <0.05 and a 95% confidence interval (CI) indicating statistically significant differences.

## RESULTS

### General clinical information

**Figure 1:**
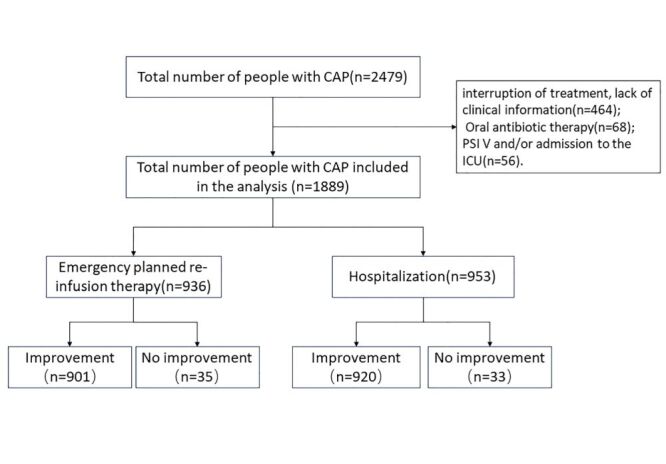
Inclusion and grouping flowcharts for emergency planned re-infusion therapy and hospitalisation in patients with CAP. CAP – community-acquired pneumonia, ICU – intensive care unit, PSI – pneumonia severity index.

The study included 2479 patients (**Figure 1**). After excluding 464 individuals who were reluctant, interrupted treatment, or were missing substantial clinical information, 68 who were treated solely with oral antibiotics, and 56 patients with PSI V pneumonia who were hospitalised in the ICU, a total of 1889 patients met the study criteria. General information of patients, such as genders, ages, and vital signs are presented in Table S1 in the **Online Supplementary Document**. The mean age was 50.95 ± 16.12 years, including 1044 (55.3%) males and 1371 (72.6%) patients under the age of 65. In terms of gender and age, males and older patients predominated in the hospitalisation group, showing a statistically significant difference (*P* < 0.05). Vital signs were generally stable among patients, with no statistical differences (*P* > 0.05) between the emergency planned re-infusion therapy group and the hospitalisation group in terms of systolic blood pressure (135.15 ± 21.37 vs. 137.11 ± 25.87), diastolic blood pressure (81.39 ± 13.97 vs. 81.08 ± 16.55), and temperature (37.29 ± 1.04 vs. 37.28 ± 1.14). However, statistically significant differences were found in pulse rate (97.54 ± 17.87 vs. 103.40 ± 20.99) and respiration rate (17.64 ± 2.04 vs. 19.21 ± 3.76, *P* < 0.001).

Laboratory test results showed that the white blood cell count, neutrophil count, and lymphocyte count in the emergency planned re-infusion therapy group were significantly lower than those in the hospitalisation group (*P* < 0.001), while the red blood cell volume was significantly higher (39.81 ± 5.47 vs. 37.74 ± 6.64, *P* < 0.001). There were no statistically significant differences in platelet count (236.75 ± 74.87 vs. 241.47 ± 94.32) and monocyte count (6.21 ± 2.41 vs. 5.96 ± 3.48, *P* > 0.05). Furthermore, levels of C-reactive protein, creatinine, blood urea nitrogen, random blood glucose, blood sodium, serum potassium, and glutamic oxaloacetic transaminase were significantly lower in the emergency planned re-infusion therapy group compared to the hospitalisation group, indicating statistically significant differences (*P* < 0.05).

### Effects of underlying diseases on emergency planned re-infusion therapy and hospitalisation in patients with CAP

Data on underlying diseases (**Table 1**) indicated that the prevalence of hypertension, diabetes mellitus, coronary heart disease, cerebrovascular disease, chronic obstructive pulmonary disease, and cancer were all lower in the emergency planned re-infusion therapy group compared to the hospitalisation group (*P* < 0.05). However, there were no significant differences in the incidence of congestive heart failure, liver and kidney diseases between the two groups (*P* > 0.05). Furthermore, 76.7% of the study subjects were classified as PSI grades I–II, 17.4% as grade III, and 5.9% as grade IV. The proportions of patients in each PSI grade were significantly lower in the emergency planned re-infusion therapy group than in the hospitalisation group (*P* < 0.001).

**Table 1:** Underlying diseases and PSI classification of patients with CAP

Underlying diseases	Total (n = 1889)	Emergency planned re-infusion therapy (n = 936)	Hospitalisation (n = 953)	*P*-value
HT*	194 (10.27%)	61 (6.22%)	133 (13.96%)	<0.001
DM*	148 (7.83%)	38 (4.04%)	110 (11.54%)	<0.001
CHD*	59 (3.12%)	15 (1.60%)	44 (4.62%)	<0.001
CVD*	45 (2.38%)	11 (1.18%)	34 (3.57%)	<0.001
CHF*	23 (1.22%)	8 (0.85%)	15 (1.57%)	0.208
COPD*	134 (7.09%)	25 (2.67%)	109 (11.44%)	<0.001
Cancer*	68 (3.60%)	7 (0.75%)	61 (6.40%)	<0.001
Liver disease*	26 (1.38%)	8 (0.85%)	18 (1.89%)	0.054
Renal disease*	32 (1.69%)	11 (1.18%)	21 (2.20%)	0.083
PSI				
*I–II*	1449 (76.71%)	809 (84.43%)	640 (67.16%)	<0.001
*III*	329 (17.42%)	101 (10.79%)	228 (23.92%)	
*IV*	111 (5.87%)	26 (2.78%)	85 (8.92%)	

CAP – community-acquired pneumonia, CHD – coronary heart disease, CHF – congestive heart failure, COPD – chronic obstructive pulmonary disease, CVD – cerebrovascular disease, DM – diabetes mellitus, HT – hypertension, PSI – pneumonia severity index

*The number of patients of emergency planned re-infusion therapy and hospitalisation.

### Factors for CAP patients' choice of emergency replanning for revisit treatment or hospitalisation

**Figure 2:**
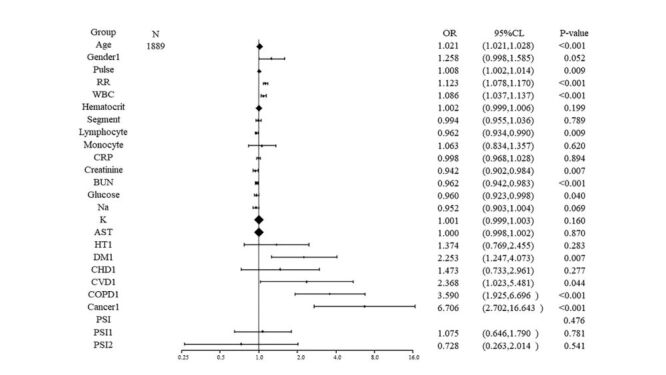
Binary outcome logistic analysis of factors affecting hospitalisation in patients with CAP. ALT – glutamic pyruvic transaminase, AST – glutamic oxaloacetic transaminase, BUN – blood urea nitrogen, CAP – community-acquired pneumonia, CHD – coronary heart disease, CI – confidence interval, COPD – chronic obstructive pulmonary disease, CRP – C-reactive protein, CVD – cerebrovascular disease, DM – diabetes mellitus, HT – hypertension, K – serum potassium, Na – blood sodium, OR – odds ratio, PSI – pneumonia severity index, RR – respiration rate, WBC – white blood cell count.

Logistic regression analysis was performed on indicators that showed statistically significant differences (**Table 2**) to identify factors influencing the hospitalisation of patients with CAP, and results were visualised in a forest plot (**Figure 2**). The analysis identified age, respiration rate, and pulse rate as independent risk factors for hospitalisation. Additionally, high blood urea nitrogen, high neutrophil counts, low blood sodium, low lymphocyte counts, and low red blood cell volume were also independent risk factors. Patients with a history of diabetes mellitus, cerebrovascular disease, pulmonary disease, and cancer were found to be more likely to require hospitalisation.

**Table 2:** Multi-level logistic analysis was performed on the outcome of the factors affecting hospitalisation of patients with CAP

Parameter (n = 1889)	OR	95% CI	*P*-value
Age	1.021	(1.021–1.028)	<0.000
Sex	1.258	(0.998–1.585)	0.052
Pulse rate	1.008	(1.002–1.014)	0.009
RR	1.123	(1.078–1.170)	<0.000
WBC	0.998	(0.968–1.028)	0.894
Hematocrit	0.962	(0.942–0.983)	<0.001
Segment	0.96	(0.923–0.998)	0.040
Lymphocyte	0.942	(0.902–0.984)	0.007
Monocyte	0.952	(0.903–1.004)	0.069
CRP	1.001	(0.999–1.003)	0.160
Creatinine	1	(0.998–1.002)	0.870
BUN	1.086	(1.037–1.137)	<0.001
AST	1.002	(0.999–1.006)	0.199
Glucose	0.994	(0.955–1.036)	0.789
Na	0.962	(0.934–0.990)	0.009
K	1.063	(0.834–1.357)	0.620
HT	1.374	(0.769–2.455)	0.283
DM	2.253	(1.247–4.073)	0.007
CHD	1.473	(0.733–2.961)	0.277
CVD	2.368	(1.023–5.481)	0.044
COPD	3.590	(1.925–6.696)	<0.000
Cancer	6.706	(2.702–16.643)	<0.000
PSI			0.476
PSI (1)	1.075	(0.646–1790)	0.781
PSI (2)	0.728	(0.263–2.014)	0.541

ALT – glutamic pyruvic transaminase, AST – glutamic oxaloacetic transaminase, BUN – blood urea nitrogen, CAP – community-acquired pneumonia, CHD – coronary heart disease, CI – confidence interval, COPD – chronic obstructive pulmonary disease, CRP – C-reactive protein, CVD – cerebrovascular disease, DM – diabetes mellitus, HT – hypertension, K – serum potassium, Na – blood sodium, OR – odds ratio, RR – respiration rate, WBC – white blood cell count, PSI – pneumonia severity index

### Treatment outcomes of emergency replanning for revisit treatment or hospitalisation

Post-treatment evaluation revealed that 889 individuals in the emergency planned re-infusion therapy group showed improvement, achieving an overall improvement rate of 96.26%, which was not statistically different (*P* > 0.05) from the 99.69% improvement rate observed in the hospitalisation group (**Table 3**). Specifically, the improvement rates for CAP-rated PSI I–II patients were comparable between the emergency planned re-infusion therapy group and the hospitalisation group (99.51 vs. 99.69%). For PSI III patients, the improvement rates were 84.16 and 89.82% in the emergency planned and hospitalisation groups, respectively, without a statistically significant difference (*P* > 0.05). Sixteen PSI III CAP patients in the emergency planned group, who did not experience relief, opted for hospitalisation and were eventually discharged. The improvement rate for PSI IV in the emergency planned re-infusion therapy group was only 50%, significantly lower than the 90.59% observed in the hospitalisation group (*P* < 0.001).

**Table 3:** Prognosis, treatment days and cost analysis table of patients with emergency planned re-infusion and hospitalisation for CAP

Parameter	Emergency planned re-infusion therapy (n = 936)	Hospitalisation (n = 953)	*P*-value
PSI I–II*	805 (99.51%)	638 (99.69%)	0.458
PSI III*	85 (84.16%)	203 (89.82%)	0.154
PSI IV*	13 (50%)	77 (90.59%)	<0.001
PSI I–IV*	901 (96.26%)	920 (96.54%)	0.805
Total days (95% CI)	5.13 ± 1.65 (5.01–5.23)	7.60 ± 3.93 (7.18–7.48)	<0.001
Total cost of treatment in CNY (95% CI)	1921.57 ± 923.16 (1866.88–1979.58)	9083.80 ± 3583.55 (8852.33–9299.01)	<0.001
Average daily treatment cost CNY/ days (95%CI)	396.66 ± 197.93 (384.56–409.23)	1252.62 ± 348.84 (1231.50–1275.10)	<0.001

CAP – community-acquired pneumonia, CI – confidence interval, PSI – pneumonia severity index

*The proportion of patients who improved in the emergency planned re-infusion therapy and hospitalisation.

The duration of treatment averaged 5.13 ± 1.65 days (95% CI = 5.01–5.23) in the emergency planned re-infusion therapy group and 7.60 ± 3.93 days (95% CI = 7.18–7.48) in the hospitalisation group, with a statistically significant difference (*P* < 0.001). The total cost of treatment was 1921.57 CNY ± 923.16 (95% CI = 1866.88–1979.58) for the emergency planned re-infusion therapy group and 9083.80  CNY ± 3583.55 (95% CI = 8852.33–9299.01) for the hospitalisation group, also showing a statistically significant difference.

## DISCUSSION

The study included 1889 patients to undergo either planned re-infusion therapy or hospitalisation, out of a total of 2479 patients with CAP (**Figure 1**; Table S1 in the **Online Supplementary Document**). It analysed the effects of vital signs, test results, and medical histories (**Table 2**; Table S1 in the **Online Supplementary Document**), followed by dichotomous outcome multilevel logistic regression analyses (**Table 2**) and the creation of a forest plot (**Figure 2**). The treatment improvement rate for patients with emergency planned re-infusion therapy was 94.98% (**Table 3**), comparable to the 96.54% improvement rate observed in the hospitalised group (*P* > 0.05). However, both the cost and duration of treatment were significantly lower in the emergency planned re-infusion therapy group (*P* < 0.001). Emergency planned re-infusion therapy could reduce the cost to about 21% of hospitalisation. Emergency planned re-infusion therapy could reduce duration of treatment to about 71% of hospitalisation. These results suggest that emergency planned re-infusion therapy has the potential to be further developed and applied as an effective means to manage CAP, reducing both the time and cost of treatment for patients.

The proportion of males with acquired pneumonia was higher than that of females, a finding consistent with previous studies which may relate to the higher percentage of male patients who smoke [15–17]. However, among those opting for emergency planned re-infusion therapy, a greater number of female patients chose this treatment, possibly because it allows them more time to care for their families. Patients over 65 years of age, who often have one or more underlying conditions such as hypertension, diabetes mellitus, and coronary artery disease, typically had higher PSI scores, PSI risk grades, and altered mental status upon admission [18–20]. For safety reasons, these patients are more likely to choose or be advised to opt for hospitalisation during consultations, resulting in a higher percentage of CAP patients over 65 being hospitalised. It was observed that patients with CAP often presented at the upper limit of regional outliers for blood pressure, pulse, respiration, and temperature, likely due to an increased willingness to seek hospital care early in the course of the disease during the COVID-19 pandemic. Patients in the emergency planned re-infusion therapy group showed no significant differences from the hospitalisation group in terms of blood pressure and temperature, but those with a rapid heart rate and fast respiration were more likely to opt for hospitalisation. The possible reason is that fast heart rate and fast breathing are more likely to cause acute organ dysfunction in patients [21]. The study also noted that white blood cell counts and C-reactive protein levels were elevated in patients with CAP, aligning with findings from previous studies [22]. However, the results of the multilevel logistic regression analysis indicated that these factors were not independent risk factors for hospitalisation.

In the study, about one-third of the patients with CAP had one or more underlying diseases simultaneously. However, it has been shown that a history of diabetes mellitus, cerebrovascular disease, tumors, and chronic obstructive pulmonary disease are independent risk factors for hospitalisation. Most patients with CAP and concurrent diabetes mellitus did not exhibit typical clinical symptoms such as polydipsia, persistent polyuria, or fluctuations in blood glucose levels, leading to an underestimation of the impact of diabetes on CAP. Previous studies have indicated that patients with CAP and diabetes experience significant blood glucose fluctuations, creating a high-glycemic environment that facilitates bacterial growth and proliferation, which complicates the treatment of pneumonia [23]. Additionally, numerous studies, systematic reviews, and meta-analyses have noted that diabetic patients are at an increased risk of developing CAP [24–27]. Patients are generally more inclined to opt for hospitalisation when CAP is accompanied by cerebrovascular disease, malignant tumors, and chronic obstructive pulmonary disease, as these conditions are associated with a poorer prognosis [28–30].

In the study, most of the patients with PSI grades of I and II (76.71%) were mildly ill and 66% of these patients returned for planned infusions in the emergency department, achieving good therapeutic outcomes. However, 44% of patients with mild illnesses preferred hospitalisation [31], possibly due to greater medical insurance reimbursement for hospitalised patients compared to outpatients with the same condition. The efficacy of treatment for PSI I and II patients through emergency planned re-infusion therapy (with an improvement rate of 99.38%) was comparable to hospitalisation in terms of clinical cure. There was no recurrence of symptoms during respiratory clinic reviews after one week, offering a safe and effective new treatment modality for CAP patients, a topic seldom addressed in previous studies. Of the patients with PSI III, 85 were clinically cured through emergency planned re-infusion therapy, achieving an improvement rate of 84.16%, which was not statistically different from the hospitalisation group (improvement rate of 89.82%) (*P* > 0.05). Sixteen patients opted for hospitalisation after three days of emergency planned re-infusion therapy due to insufficient relief but were eventually discharged with improved symptoms. These 16 patients experienced fewer subsequent hospitalisations compared to those in the hospitalisation group, suggesting that emergency planned infusion therapy can shorten hospital stays, a finding that warrants verification in future clinical studies. For patients with PSI IV, 13 patients (50%) were transferred to hospitalisation after emergency planned re-infusion therapy, and four of these patients experienced further progression of their condition, necessitating transfer to the ICU for intensive care. In comparison, eight patients (9.41%) with PSI IV in the hospitalisation group showed no improvement; of these, three were transferred to the ICU, four opted for transfer treatment, and one chose to discontinue treatment and was automatically discharged from the hospital. For PSI IV patients, emergency planned re-infusion therapy was not as effective as inpatient treatment. We speculated that the reason may be the lack of rehabilitation treatment in emergency planned re-infusion therapy (hospitalisation included rehabilitation treatment). Hence, hospitalisation is recommended and should be mandatory for patients in the high-risk PSI IV group to reduce the rates of severe complications and mortality in CAP [32].

The average treatment duration was five days for emergency planned re-infusion therapy patients and seven days for hospitalised patients. A study by Dunbar LM et al. found no difference in the efficacy of short-course (3–5 days) and long-course (seven days) treatments in terms of clinical cure and recurrence rates [33–35], suggesting that the duration of antibiotic use in emergency planned re-infusion therapy is both effective and reasonable. The study also revealed that emergency planned re-infusion therapy significantly reduced costs by approximately two-thirds compared to hospitalisation, offering an optimal balance of improved clinical outcomes and cost savings for financially disadvantaged patients.

**Figure 3:**
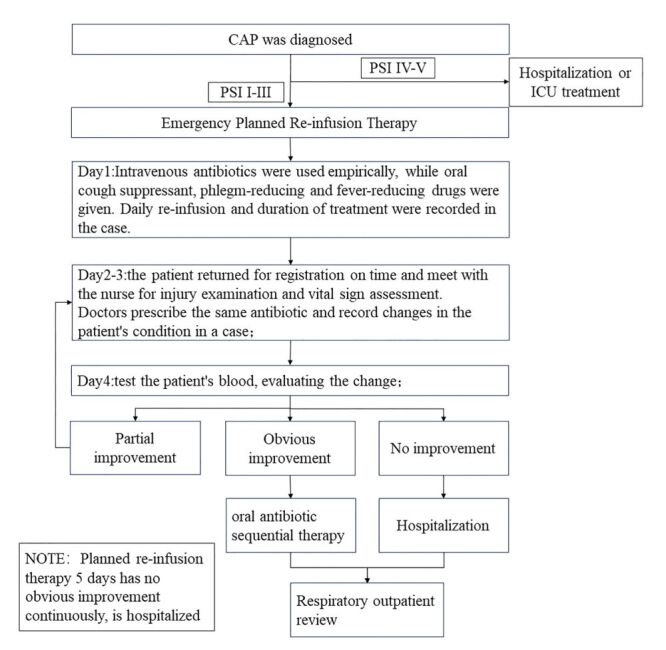
Procedure standard for emergency planned re-infusion therapy for patients with community-acquired pneumonia. CAP – community-acquired pneumonia, ICU – intensive care unit, PSI – pneumonia severity index.

Community-acquired pneumonia accounts for 5–12% of all cases of lower respiratory tract infections in adults and significantly increases direct medical costs, particularly those associated with hospitalisation [36,37]. Therefore, optimising the treatment of CAP represents a significant public health consideration. The study summarised the recommendations for emergency planned re-infusion therapy for patients with CAP PSI I–IV, which continued to be practically applied in the department as shown in **Figure 3**. On the first day, patients were prescribed intravenous antibiotics and oral medications to relieve cough, eliminate phlegm, and reduce fever based on their condition. Patients were informed of the daily return time for infusion and the duration of treatment, which were recorded in the chart. From the second day, patients needed to regularly return to the clinic to register, meet with the nurse for injury examination and vital sign assessment. Doctors continued to prescribe antibiotics and record changes in the patient's condition. After three days of infusion, patients were to undergo blood tests, with the results and changes in clinical symptoms provided to doctors for assessment of improvement. Patients showing improvement were prescribed sequential oral antibiotic therapy and scheduled for follow-up in the respiratory clinic, while those without remission were hospitalised. It is important to monitor patients' vital signs, condition improvements, and timely review of abnormal test indicators during daily planned returns for infusion, especially for those rated as PSI IV. Patients showing worsening conditions should be strongly advised to hospitalise to minimise the risk of death. Patients rated as PSI I–III should transition to oral antibiotic sequential therapy after a short-term (3–5 days) of effective antibiotic therapy, which has been proven reasonable and effective in previous studies without increasing patient risk [38,39]. Lastly, patients discharged after emergency infusion should have a follow-up visit scheduled at a respiratory clinic to prevent recurrence.

The aging of the global population is gradually increasing, and the proportion of people over 65 years old will account for an increasing proportion, and they are the high-risk groups for CAP. This study may provide a less time-consuming and less costly treatment option for patients with PSI I–III, but more data on this patient population is needed in the future to verify the feasibility of this option. It is suggested that future research and analysis of cost-related benefits, such as potential savings from health care systems or patient out-of-pocket costs, could be strengthened. This study mainly focused on the Chinese population, and it is suggested that similar studies can be conducted in different countries in the future for comparison. It is also the direction of research whether the emergency planned re-infusion therapy for CAP can be applied to other diseases. In summary, CAP planned re-infusion therapy is a valuable treatment method that can reduce both treatment costs and duration, providing patients with additional treatment options.

### Limitations

This study still has the following four limitations. The first limitation was the lack of patients under 14 years of age in this study. Then there was methodological limitation, and there may still be underlying diseases that cannot be collected in this study. Finally, the study lacked an outpatient treatment group, so this study could not compared the differences in financial burden between outpatient, emergency, and inpatient care.

## CONCLUSIONS

Emergency planned re-infusion therapy for CAP is an effective and cost-efficient alternative that can reduce both treatment duration and costs, particularly for patients with PSI I–III. It is recommended that emergency physicians give priority to emergency re-infusion therapy for patients with PSI I–III. In addition, it is recommended that hospitals strengthen the classification and treatment training programmes for emergency department physicians to recognise the patients with PSI I–IV.

## Additional material


Online Supplementary Document
