# Supplementary material for: Emergency planned re-infusion therapy and hospitalisation for community-acquired pneumonia: a retrospective case-control study
Source: J Glob Health. 2025 Jan 31;15:04044. doi: 10.7189/jogh.15.04044 (PMC11783337; doi:10.7189/jogh.15.04044)
Supplement: Online Supplementary Document [file jogh-15-04044-s001.pdf]

Table S1. General data sheet for emergency planned re-infusion therapy and hospitalisation in patients with CAP

**Parameter**	**Total (n = 1889)**	**Emergency planned re-infusion therapy (n = 936)**	**Hospitalisation (n = 953)**	***P*-value**
Male	1044 (55.27%)	486 (51.92%)	558 (58.55%)	0.004
Female	845 (44.73%)	450 (48.08%)	395 (41.45%)	
Age < 65	1371 (72.58%)	791 (84.51%)	580 (60.86%)	＜0.001
Age ≥ 65	518 (27.42%)	145 (15.49%)	373 (39.14%)	
BPS (mmHg)		135.15 ± 21.37	137.11 ± 25.87	0.074
BPB (mmHg)		81.39 ± 13.97	81.08 ± 16.55	0.658
Temperature		37.29 ± 1.04	37.28 ± 1.14	0.913
Pulse rate		97.54 ± 17.87	103.40 ± 20.99	＜ 0.001
RR		17.64 ± 2.04	19.205 ± 3.76	＜ 0.001
WBC		9.80 ± 4.18	10.76 ± 7.07	＜ 0.001
Hematocrit		39.81 ± 5.47	37.74 ± 6.64	＜ 0.001
Platelets		236.75 ± 74.87	241.47 ± 94.32	0.229
Segment		73.01 ± 12.23	76.31 ± 14.57	＜ 0.001
Lymphocyte		19.14 ± 10.54	15.08 ± 9.94	＜ 0.001
Monocyte		6.21 ± 2.41	5.96 ± 3.48	0.067
CRP		43.02 ± 48.47	69.05 ± 87.40	＜ 0.001
Creatinine		73.55 ± 38.52	87.84 ± 84.15	＜ 0.001
BUN		4.62 ± 2.54	6.13 ± 5.61	＜ 0.001
Glucose		7.04 ± 2.61	7.78 ± 2.98	＜ 0.001
Na		137.03 ± 3.56	135.45 ± 4.68	＜ 0.001
K		3.70 ± 0.37	3.76 ± 0.54	0.003
AST		33.74 ± 19.57	38.61 ± 43.67	0.002
ALT		29.16 ± 25.34	28.09 ± 30.64	0.409

ALT – glutamic pyruvic transaminase, AST – glutamic oxaloacetic transaminase, BPB – diastolic blood pressure, BPS – systolic blood pressure, BUN – blood urea nitrogen, CAP – community-acquired pneumonia, CRP – C-reactive protein, K – serum potassium, Na – blood sodium, RR – respiration rate, WBC – white blood cell count
